# Use of Surface EMG in Clinical Rehabilitation of Individuals With SCI: Barriers and Future Considerations

**DOI:** 10.3389/fneur.2020.578559

**Published:** 2020-12-18

**Authors:** Rakesh Pilkar, Kamyar Momeni, Arvind Ramanujam, Manikandan Ravi, Erica Garbarini, Gail F. Forrest

**Affiliations:** ^1^Center for Mobility and Rehabilitation Engineering Research, Kessler Foundation, West Orange, NJ, United States; ^2^Department of Physical Medicine and Rehabilitation, Rutgers – New Jersey Medical School, Newark, NJ, United States; ^3^Tim and Caroline Reynolds Center for Spinal Stimulation, Kessler Foundation, West Orange, NJ, United States; ^4^Koneksa Health, New York, NY, United States

**Keywords:** spinal cord injury (SCI), electromyography (EMG), EMG barriers, biomedical signal processing, clinical rehabilitation, high-density EMG

## Abstract

Surface electromyography (sEMG) is a widely used technology in rehabilitation research and provides quantifiable information on the myoelectric output of a muscle. In this perspective, we discuss the barriers which have restricted the wide-spread use of sEMG in clinical rehabilitation of individuals with spinal cord injury (SCI). One of the major obstacles is integrating the time-consuming aspects of sEMG in the already demanding schedule of physical therapists, occupational therapists, and other clinicians. From the clinicians' perspective, the lack of confidence to use sEMG technology is also apparent due to their limited exposure to the sEMG technology and possibly limited mathematical foundation through educational and professional curricula. Several technical challenges include the limited technology-transfer of ever-evolving knowledge from sEMG research into the off-the-shelf EMG systems, lack of demand from the clinicians for systems with advanced features, lack of user-friendly intuitive interfaces, and the need for a multidisciplinary approach for accurate handling and interpretation of data. We also discuss the challenges in the application and interpretation of sEMG that are specific to SCI, which are characterized by non-standardized approaches in recording and interpretation of EMGs due to the physiological and structural state of the spinal cord. Addressing the current barriers will require a collaborative, interdisciplinary, and unified approach. The most relevant steps could include enhancing user-experience for students pursuing clinical education through revised curricula through sEMG-based case studies/projects, hands-on involvement in the research, and formation of a common platform for clinicians and technicians for self-education and knowledge share.

## Introduction

The current state-of-the-art rehabilitation for individuals with spinal cord injuries (SCI) utilizes technologies such as neuromodulation using exoskeleton robotics, functional electrical stimulation (FES), treadmill training with and without body-weight support (BWS) in addition to the traditional exercise-based rehabilitation. The recent developments in neurorehabilitation research and technologies have resulted in a shift in focus toward the recovery of function through high intensity repetitive training after SCI (1). Some of the technologies such as epidural or transcutaneous spinal stimulation, robotic exoskeletons are currently under investigation while techniques such as FES (e.g., FES cycling, rowing) (2) and treadmill training using BWS (3) are commonly used in clinics to assist with the functional tasks such as respiration, mobility, hand function, metabolism, bladder, bowel or sexual function (2, 4, 5). Irrespective of the intervention approach used, the functional status as well as the evolutions of motor impairments and motor recovery are often tracked by visual and manual assessments in the clinic. To date, the primary method for evaluating the motor function for SCI is the American Spinal Injury Association Impairment Scale (AIS), which tests manual muscle strength in five key muscles in each limb and examines sensory function (6). Although easy to perform, such approaches are subjective and not sensitive to understanding the changes at the neuromuscular levels. Particularly for the interventions that target the neuromuscular mechanisms via application of electrical stimulation to the nerves, or peripheral musculature, the objective and quantifiable information on the myoelectric output of targeted muscles is highly relevant. For instance, when a clinician uses FES -the technique that involves the application of electrical current to the neuromuscular junction and cause contractions in paralyzed muscles (7)- it is clinically desirable to evaluate the resultant myoelectric output of the stimulated muscle. The questions such as “is the stimulation intensity sufficient to induce the desired contraction for the targeted movement?” “is the stimulation causing the targeted muscle to fatigue?” or “are the selected parameters appropriate for the patient to perform the desired task?” become highly relevant to the clinician to deliver patient-specific and effective interventions. Such questions are highly significant for any intervention that targets mobility and motor rehabilitation.

Surface electromyography (sEMG), a non-invasive technique for assessing the myoelectric output of a muscle, can provide objective answers to these significant questions. sEMG has shown great promise in neurorehabilitation research and has been a widely-utilized tool to assess neuromuscular outcomes in research (8). However, the application of sEMG in a clinical environment has been limited (9). The clinicians' perspectives on the use of sEMG have reported several barriers including limited time and resources, clinically inapplicable sEMG system features and the majority of clinicians' lack of training and/or confidence in utilization of sEMG technology (10, 11). In the domain of the SCI population, in addition to the aforementioned challenges of using sEMG in the clinic, severely impaired physiological and structural state of the spinal cord after SCI (compared to other pathologies such as stroke, traumatic brain injury, multiple sclerosis, etc.) further limits sEMG usage to provide time-efficient, meaningful interpretations. In this perspective report, we discuss these barriers and the directions toward overcoming these limitations that hinder the widespread use of sEMG technology in the clinical rehabilitation of individuals with SCI.

## Barriers in the Use of sEMG in SCI neurorehabilitation

### General Barriers to Use sEMG in a Clinical Setting

Several barriers can be identified that restrict the adoption of sEMG technology in a clinical environment.

#### Lack of Information at Motor Unit (MU) Level

Needle EMG (nEMG) and fine wire EMG (fwEMG) are the invasive forms of EMG for accessing neurophysiological attributes of neuromuscular diseases. However, the invasiveness, discomfort, and limited applicability of these techniques on multiple muscles during dynamic tasks limit their use in the clinic. Nonetheless, nEMG still is gold standard for clinical diagnosis of nerve and muscle pathologies and preferred over non-invasive sEMG (12) for neurophysiological applications. This is because of the limited spatial resolution of sEMG that results in poor fidelity recordings of high-frequency signals (e.g., polyphasic potentials, fibrillation potentials, and positive sharp waves) (12). In addition, the electrical cross-talk between two or more neighboring muscles restricts the sEMG to identify the origin of the electrical signal when these muscles are active simultaneously (12). Further, the sEMG recorded from a muscle does not yield a non-ambiguous extraction of single MU information. As a result, the report of the therapeutics and technology assessment subcommittee of the American Academy of Neurology reported the sEMG technique unusable for clinical neurophysiological purposes (13). While the bipolar sEMG is used to measure muscle activations, the advent of high-density surface EMG (HDEMG) has made the extraction of MU features possible (14–16). Availability of such a sensitive tool is even more significant for individuals with clinically diagnosed motor and sensory complete SCI who do not have intact reflexes and who may still have intact neuronal axons across the injury lesion (17). However, in order to accomplish this, a careful application of sEMG decomposition and expertise in signal acquisition, interpretation of results, and manual assessment of decomposition quality is required (14–16). Further, the examination of the Motor Unit Number Index (MUNIX) in paralyzed muscles has been implemented to monitor MU loss after SCI (18). However, this approach requires intense experimental and computational setup, and specific selection criteria which may not be clinically feasible.

#### Lack of Available Time for a Clinician

**Figure 1:**
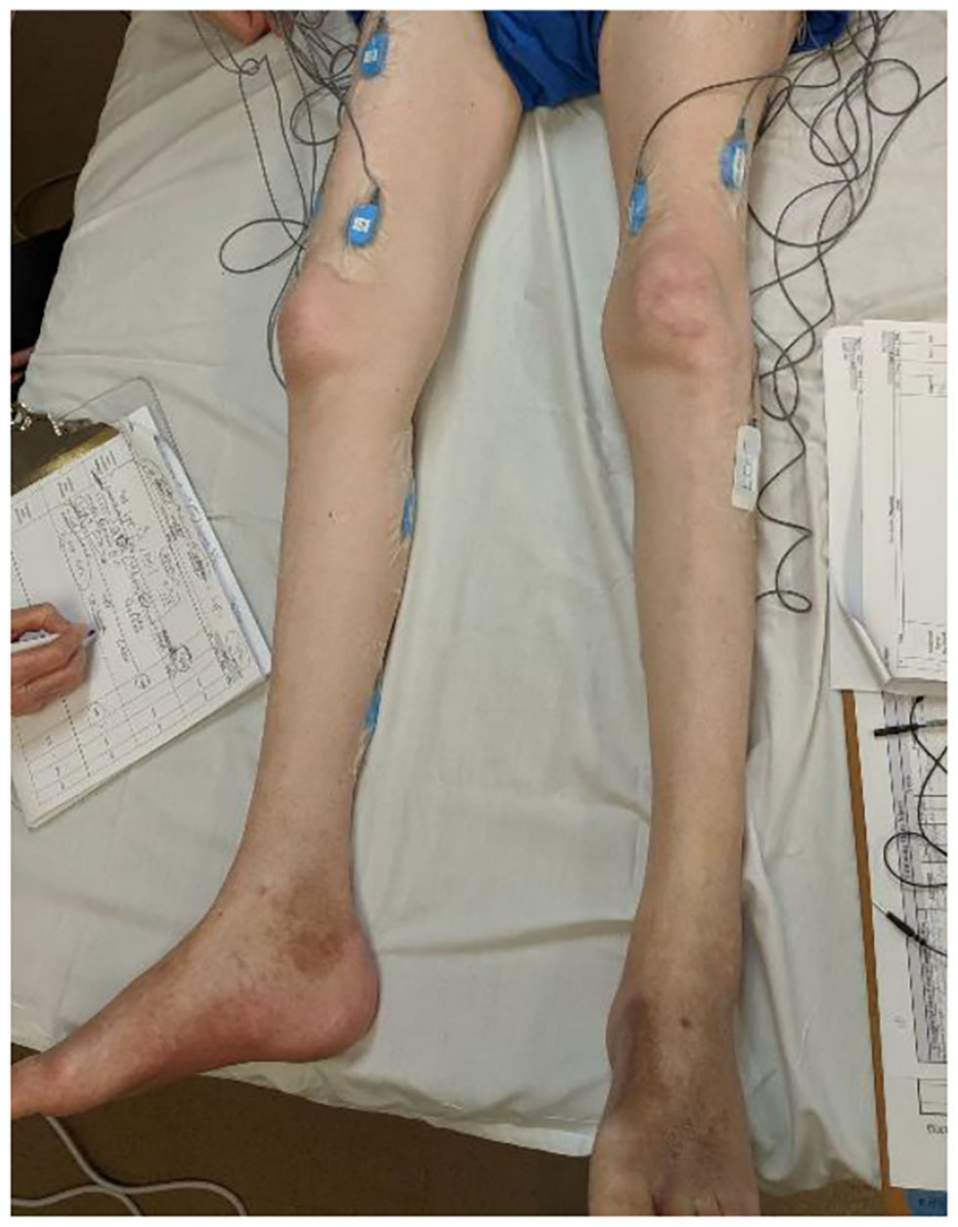
An EMG set up for assessing neuromuscular responses prior to a rehabilitation intervention for an individual with SCI.

A study collected perspectives of 22 clinicians [physical therapists (PT), occupational therapists (OT), and physiatrists] and reported limited clinician time as one of the barriers to the uptake of sEMG technology in clinics (11). The time-consuming aspect of sEMG technology presents a significant barrier to its translation into clinical practices. Electrodes and skin preparations, electrode placements, equipment setup, collecting maximal volitional contractions (MVC) for normalization prior to recording the data during activities of interest take significant time. Figure 1 illustrates the sEMG placements for recording of lower extremity responses from an individual with an SCI. Balancing a busy work schedule has been reported as one of the barriers to caring for patients, particularly for novice PTs (19). Therefore, the acceptance of sEMG technology that requires significant prep-time is low as the added time could adversely affect PT performance and care in the clinic.

#### Limited Background and Training Through Professional Curricula

Most of the PT and OT programs offer wide-ranging coursework in the rehabilitation domain including human anatomy, neuroscience, biomechanics, kinesiology, movement analysis, evidence-based practice, pharmacological interventions, etc. Irrespective of the breadth of topics covered, there is minimal focus on the technological aspects of rehabilitation. As a result, rehabilitation tools such as sEMG are theoretically taught, but practical knowledge imparted is limited. Further, the educational content may not cover ever-evolving aspects of sEMG technology and its applications. Feldner et al. reported that many clinicians felt less confident to use sEMG in clinics due to their limited experience (11). According to the study, newer clinicians pointed the “need for practice” and the seasoned clinicians weren't “tech savvy,” making the clinical adoption of sEMG technology difficult (11). However, one limitation of this survey was the limited geographical spread of the clinicians who participated as they were recruited from rehabilitation settings within the Seattle metropolitan area (WA, USA). In a more recent survey by Manca et al., 35 EMG experts from different educational, professional and geographical backgrounds supported the clinical utility of sEMG for optimizing the quantification of muscle and physical function, to define the intervention plan, and optimize other methods used to quantify muscle and physical function (20). However, the collective opinion of these experts also confirmed the utilization of sEMG was more common in technical/methodological research than clinical research (20). The barriers that prevent prompt transfer of sEMG into practice were reported to be slow dissemination of research findings and the lack of education on sEMG (20). Further, successful adoption of any technology in the clinic not only involves collecting the information/data but also helps in making data-driven clinical decisions in functional diagnosis, recommending appropriate interventions, and optimizing the rehabilitation outcomes. In terms of sEMG, the processing and interpretation of the data require a multidisciplinary approach. This involves the working knowledge of several technical domains such as instrumentation, signal processing and analysis, algorithm development, and statistical analyses. The availability of such expertise can be challenging in a clinical setting. Identifying the experts with such a skillset and establishing collaborations could be time-consuming, and impractical for daily-workflow at the clinic. If a clinician wants to gain the necessary working knowledge on sEMG technology, there is no centralized knowledge-base where clinicians can, not only develop their understanding of sEMG procedures and data analyses but also interact with other clinicians and researchers in this specific domain to share ideas, discuss outcomes and even collaborate at the institutional levels. The training and education of teachers who are educating future clinicians is another important factor. In many countries where there are no doctoral-level programs in rehabilitation or physiotherapy, there is a scarcity of academic professors with doctorate-level credentials. Therefore, the educational experience of students in such countries may lack rigor, practical exposure to the technology, and the state-of-the-art information on sEMG practices and guidelines.

#### Lack of Technology Transfer From Research to Clinic

The field of sEMG is always evolving and new algorithms for sEMG processing, analysis, and classifications are continuously being developed. However, the rate at which these technological advances are frequently integrated into the existing sEMG systems is limited. For instance, many of the existing off-the-shelf sEMG systems have not gone beyond implementing the basic sEMG features such as mean and root-mean-square (RMS) amplitudes, moving average or RMS envelopes, basic filtering and rectifications, and basic Fourier-based analysis. Automatic burst or ON-OFF detections, activation timing analyses, signal decomposition, and time-frequency analyses are widely published (21–25) and accepted EMG analysis techniques that have not been integrated into most of the commercial systems; as a result, these techniques have not been transferred from research to clinic. This issue stems from lack of education or training on the application of such analysis methods in a clinical setting, resulting in virtually no demand for a commercial EMG system with these capabilities, which in turn creates an insignificant market to manufacture such EMG devices. Therefore, the absence of commercial pressure further limits the development of said devices and education of operators to ultimately transfer research findings into the clinic.

#### Institutional Level Barriers

In addition to the sEMG setup time, other challenges hinder the adoption of sEMG technology in the clinic. Such barriers include the functionality in multiple environments, portability, the facility layout, purchasing cost and maintenance, providing evidence to support returns on such investments, and staff training.

### Barriers Specific to the SCI Population

The need for assessing neuromuscular responses is highly significant for individuals with SCI, particularly motor complete SCI (cSCI). Studies have demonstrated the presence of intact neuronal axons across the lesion, even after cSCI (17). For instance, Calancie et al. (26) reported retained voluntary EMG control over one muscle in the foot in a small group of participants classified as motor complete. These findings highlight the significance of the ability to monitor neuromuscular responses during neuromuscular electrical stimulation (NMES) for cSCI for whom any functional and motor-related changes may not be apparent, while intrinsic electrophysiological changes and residual volitional neuromuscular drive may still be present. In evaluating the efficacy of any clinical therapy, the effects may not be visible at the functional or biomechanical levels but changes could be present at the neuromuscular level. Therefore, assessing neuromuscular output is critical to optimize the effects of any rehabilitation intervention for SCI. Currently, there are no standardized procedures for processing and interpreting sEMG data specific to the cSCI population; this may have vastly contributed to the diverse sEMG interpretations and/or continued reliance on outcome measures, such as force and torque. In addition, the lack of standards for sensors, configurations, electrode placement, and recording protocols has adversely affected the possibility of its integration into routine clinical use (9). Despite the 20-year presence of the EU project on “Surface EMG for Non-Invasive Assessment of Muscles (SENIAM),” real international standards are still missing (10). The diminished or weaker sEMG signals yield limited consensus on answers to the most basic questions such as, “*is the muscle active?*” “*what is the strength of the activation?”* or more complicated ones such as, “*what is the volitional contribution and how it relates to the applied stimuli during electrically induced activations?*” Answers to such questions remain unclear as there is no standardized approach to first process and then interpret such data. The existing off-the-shelf systems are not specifically tuned to address these SCI-specific challenges. For example, the most significant barrier in using sEMG during FES is interpreting the recorded sEMG signals due to the overpowering presence of stimulation artifact. The stimulation artifact is a broadband signal with widespread stimulation frequency harmonics at high amplitudes that engulf the myoelectric responses in sEMG. Particularly when a train of ES pulses is applied, the sEMG recordings are accompanied by ES artifact spikes with magnitudes that are manifold compared to the actual MU outputs. Moreover, the presence of stimulation artifact is not confined in the time-domain; it is also observed in the frequency domain. The harmonics of stimulation frequency overlap with the majority of the energy bands in a typical sEMG frequency spectrum (20–350 Hz). As a result, traditional selected-filtering of frequency bands, to remove ES artifacts, is ineffective and results in significant data loss (23). The ES artifact affects features derived from the sEMG signal; for instance, it biases conduction velocity estimations, spectral characteristic frequencies, and M-wave amplitudes (27). In the domain of SCI rehabilitation, where ES waveforms are often delivered as bursts (train of pulses) with high intensities and wide-ranging frequencies, the resultant contamination of sEMG recordings obstructs the understanding of the direct implication of FES on the neuromuscular output in terms of activation intensity (voluntary or ES induced), MU recruitment, and muscle fatigue. This is particularly impeding in studies where FES is combined with volitional efforts that need to be monitored or modulated in real-time to achieve optimal outcomes.

## Future Directions

Rehabilitation professionals' acceptance and adoption of technologies rely on conditions that facilitate their use such as scheduling, support and a conductive environment (28). The following are the steps toward achieving these key aspects of sEMG utilization in the clinical neurorehabilitation.

### Enhancing Knowledge and User-Experience

In order to ensure all rehabilitation professionals, especially clinicians, get an early exposure to the sEMG technology, the educational and professional training programs could integrate hands-on sEMG experience through case studies or small research projects. The clinicians could also enhance their involvement in ongoing sEMG-related research activities and get exposed to the several practical aspects of sEMG through interactions with their non-clinical counterparts (e.g., engineers, technicians, data scientists). The interfaces running the EMG data collection and processing algorithms with minimal user inputs could be beneficial for their widespread implementations. Another goal could be set to successfully transfer EMG-related research products (data collection, processing and analysis algorithms) into a clinical environment. Irrespective of the programming platforms (Matlab, Python, etc.) on which these algorithms are built upon, simple user-interfaces, application programming interfaces (APIs) and/or open-source executables can be created for their unobstructive and intuitive use by the clinicians with non-technical backgrounds. A centralized knowledge-base can be used to create and disseminate the sEMG tutorials on topics ranging from the basics of sEMG technology to step-by-step guidelines for data processing. Such a centralized open-source platform can also facilitate the collaborations among investigators and sEMG users with overlapping interests. With the help of well-established societies such as International Society of Electromyography and Kinesiology (ISEK), IEEE Engineering in Medicine and Biology Society (EMBS), Society for Neuroscience (SFN), and several societies of clinical motion analysis [Gait Clinical Movement Analysis Society (GCMAS), the European Society for Movement Analysis in Adults and Children (ESMAC), Societa' Italiana di Analisi del Movimento in Clinica (SIAMOC) etc.], the long-term goal can be set to developing international scientific meetings or chapters specific to sEMG applications in specific rehabilitation domain (e.g., FES) where the specific pool of researchers can meet, share knowledge and collaborate. In recent years, efforts have been made to provide open-access tutorials and consensus articles on sEMG-related best practices, such as the consensus standards and guidelines on the sEMG detection (29), sEMG signal conditioning and preprocessing (30), and analysis of MU discharge characteristics using HDEMG (14). *The Consensus for Experimental Design in Electromyography (CEDE)* project, an international initiative which aims to guide decision-making in recording, analysis, and interpretation of sEMG have published the guidelines on the sEMG electrode selection and amplitude normalization (31, 32). Despite of these past and present efforts, these well-accepted guidelines, procedures and standards are not known to many clinicians. The paradigm shift in transferring such significant knowledge to clinic is only possible when the new generations of students pursuing education and professional training in clinical rehabilitation (e.g., PT, PTA, MPT, DPT, DScPT, PhD) are taught these “best practices in sEMG” by qualified teachers.

### EMG for Real-Time Monitoring and Biofeedback During Rehabilitation

The instantaneous quantification of muscle response can serve as an important marker to track the impairment as well as recovery during rehabilitation. With access to the EMG in real-time, the clinicians or researchers can quantify, track, and manipulate levels of voluntary efforts by modulating intervention parameters. For example, if a clinician observes that the FES frequency of 100 Hz is causing a muscle to fatigue faster with less voluntary participation (shown by EMG features such as amplitude), s(he) could change to a lower stimulation frequency, which could potentially increase voluntary contribution and reduce fatigue due to stimulation, thus making the session still productive. Such modulations could happen simply by patient's own feedback on fatigue but the data-driven nature of this decision making could make the training more objective, patient-specific, safe and less *ad hoc*. This could result in more effective interventions for better long-term benefits.

### A Ranking System for Standardization of EMG Interpretations for the SCI

Motivated by the ranking system provided by Heald et al. (17), a standardized sEMG ranking system can be developed to quantify the state of the residual neuromuscular output, especially during FES-based rehabilitation for SCI. For example, *Rank 1 – sEMG signal can be classified as no activity, baseline noise*; *Rank 2 – Sparse MU action potentials*; *Rank 3 – Burst of activity but no clear correlation to stimulation profile (e.g., FES, etc.)*; *Rank 4 – Burst of activity with partial correlation to stimulation*; *Rank 5 – Repeated burst of visible activity that is significantly correlated with applied stimulation*. Ranking procedures can be validated by visual inspection as well as automated, software-driven inspections. Such a standardized approach can track progress during or after different interventions. Once accepted and implemented, common standardized outcomes would enable comparing different interventions for efficacy.

### The Potential Impact on the Rehabilitation Costs for SCI

For many of the SCI patients, functional or motor changes may not be present but electrophysiological changes or residual voluntary muscle activations may still be present (17, 26, 33, 34). If a clinician cannot directly track the volitional efforts or functional improvements, then medical reimbursement is suspended after only a few weeks with no ultimate benefit to the participant. If sEMGs show the neuromuscular changes during an intervention for individuals with SCI with no changes in functional status, researchers and clinicians can still continue with ongoing interventions and anticipate better outcomes. On the other hand, investing in expensive interventions for several months for non-responders is a financial liability. Thus, sensitive and reliable measures of neuromuscular recovery, designed specifically for the spectrum of SCI-induced deficits can lead to long-term functional improvement that would have a dramatic impact both on the quality of life and financial liability for those suffering from SCI.

In summary, addressing the current barriers in widespread use of sEMG in SCI rehabilitation will require a collaborative, interdisciplinary, and unified approach. Nonetheless, sEMG technology has the potential to present significant opportunities that can allow clinicians and researchers to transform future interventions into effective and impactful rehabilitation modalities for individuals with SCI.

## Data Availability Statement

The data sharing will be contingent upon the regulations applied by the funding agency. Requests to access the datasets should be directed to rpilkar@kesslerfoundation.org.

## Ethics Statement

The studies involving human participants were reviewed and approved by Kessler Foundation Institutional Review Board. The patients/participants provided their written informed consent to participate in this study.

## Author Contributions

RP and KM drafted the manuscript. All authors contributed to the revisions.

## Conflict of Interest

The authors declare that the research was conducted in the absence of any commercial or financial relationships that could be construed as a potential conflict of interest.
